# Effect of Infra Low Frequency (ILF) neurofeedback training on EEG in children with autism spectrum disorders

**DOI:** 10.12669/pjms.40.7.8246

**Published:** 2024-08

**Authors:** Shemaila Saleem, Syed Hamid Habib

**Affiliations:** 1 Shemaila Saleem, MBBS, MPH, MPhil. Department of Physiology, Federal Medical College, Shaheed Zulfiqar Ali Bhutto, Medical University (SZABMU), Islamabad, Pakistan; 2 Syed Hamid Habib, MBBS, PhD, PGD, DHPE, CHR, CRSM, CME. Department of Physiology, Institute of Basic Medical Sciences, Khyber Medical University, Peshawar, Pakistan

**Keywords:** Neurofeedback, Autism Spectrum Disorders, EEG

## Abstract

**Objective:**

To investigate whether Infra-low frequency Neurofeedback (ILF-NFB) training can improve brain electrical activity in children with autism spectrum disorders ASD.

**Method:**

This single arm pre and post intervention study was carried out at IBMS (Institute of Basic Medical Sciences), Khyber Medical University, Peshawar and Shaheed Zulfiqar Ali Bhutto Medical University (SZABMU), Islamabad from January 2021 to December 2022. A purposive sampling technique was used. Thirty-five ASD children (male=24; female=11; 7-17 years) were provided with 30 sessions of infra low frequency (ILF) neurofeedback training for 15-20 minutes, during 10 weeks. Childhood Autism Rating Scale (CARS) scoring was done and electroencephalogram (EEG) activity was compared before and after ILF-NF training sessions.

**Results:**

Around 62.9% participants had mild-moderate autism and 37.1% had severe autism. Wilcoxon Signed rank test revealed a significant decline in delta (Pre-test=47.31±19.22, Post-test=22.07±6.83; p=<0.001), theta (Pre-test=24.75±16.62, Post-test=12.37±3.59; p=<0.001) and alpha (Pre-test=12.01±9.81, Post-test=4.03±1.61; p=< 0.001) waves. Mann Whitney U test exhibited no significant gender differences in EEG pattern before and after neurofeedback except in theta waves (*p*=0.03) before the intervention.

**Conclusion:**

Decline in delta, theta, beta and alpha waves propose that ILF-NF training can be effective in improving the EEG activity. ILF-NFB can be perceived as a valuable non-invasive, non-pharmacological intervention for improving EEG pattern via reintegration of brain activity resulting in increased the attention and focus, enhanced mental stability and cognitive engagement.

## INTRODUCTION

Autism Spectrum disorder (ASD) is a complex neurodevelopmental disorder and manifests in the early years of life.^1^ In autism spectrum disorder (ASD), the frequency of atypical EEG pattern ranges between 10-83 %, as reported in the literature. Abnormal EEG pattern predicts poor outcomes in terms of language, cognition and educational achievements.^2^ In people with autism, there is a general shift in the EEG spectrum, which changes the power profile. There is reduced power in the mid frequency region (alpha) and enhanced power at lower frequencies (delta, theta) and higher frequencies (beta, gamma).^3^

Neurofeedback is a form of training during which participants monitor their own brain waves via audio-video feedback to learn reinforcement and compensation.^4^ This tool for self-regulation of brain activity is used to trigger neuroplasticity. It is an efficient modality to improve electrophysiological changes of specific cortical areas of the brain.^5^ Infra-low frequency neurofeedback (ILF-NFB) involves training with ultra-low frequencies. The advantage of ILF-NFB is the rapidity with which the results are obtained. ILF-NFB explicitly accesses the functional connectivity of the intrinsic connectivity networks. These low frequencies also regulate glial-neuron system, autonomic nervous system and emotional equilibrium.^6^

Electroencephalography (EEG) mainly measures neurophysiological variations due to post synaptic activity in the neocortex. The therapeutically significant frequency ranges span from 0.3 to 100 Hz. Delta waves dominate in deep sleep and are seen in tasks of salience and attention. Theta waves are associated with memory processes. The precise timings of sensory and cognitive inhibition are associated with alpha waves, which are seen in awake, relaxed people. Beta waves are linked to motor behaviour, active task involvement, and alertness.^7^ Research has shown a raised theta and beta activity at the central and parietal lobes in ASD.^8^ Raised delta power in the frontal or fronto-central areas in ASD individuals have also been observed.^9^

Studies have assessed the influence of NFB therapy on EEG pattern in ASD participants. Post intervention theta/beta ratio in the participants decreases and revert to normal frequencies associated with a better scholastic and intellectual performance along with a reduction in challenges with focus, anxiety, aprosodias, and social functioning.^10^ A decline in symptoms of autism and improved executive functioning in accord with a decrease in theta and delta power after NFB has also been observed.^8^

In Pakistan, the inability to provide rehabilitation services during initial years of life is a source of stress for care givers and it is necessary to create realistic, affordable community-level interventions that can be incorporated into current healthcare systems.^11^ The paucity of data in Pakistan makes it difficult to estimate the prevalence of ASD in this population, identify risk factors, or even develop effective intervention strategies.^12^

Our study aimed at monitoring the real time EEG activity in ASD children and interpret the changes before and after 30 NFB sessions. The purpose of the study was to explore the impact of NFB training on EEG amongst ASD population.

## METHODS

This was a single arm Pre and Post intervention study carried out from June 2021 to June 2022 at Khyber Medical University (KMU), Peshawar and Shaheed Zulfiqar Ali Bhutto Medical University (SZABMU), Islamabad. Purposive sampling technique was utilized. A sample size of 28 was calculated by G power using an α cut-off value of 5% (0.05). Power was set at 90% (0.9). Considering 20% attrition, 42 ASD children were recruited and 35 of them were analyzed as they were able to complete 30 sessions of NFB.

### Inclusion & Exclusion Criteria:

**Fig.1:**
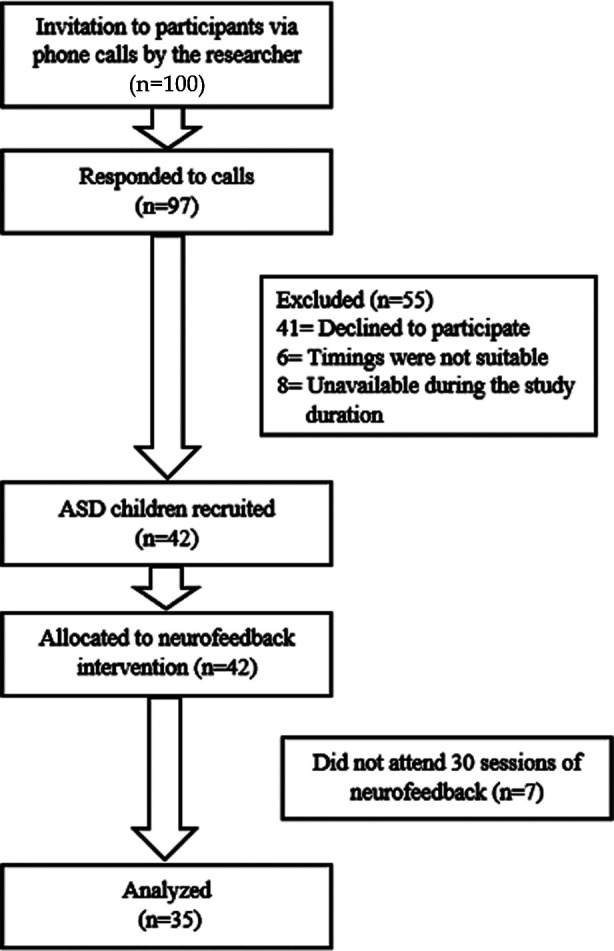
CONSORT flow diagram showing the recruitment of participants.

Recruitment of the participants was done by telephonically calling parents of ASD children. Children and Adolescents (age=7-17years) recruited (Fig.1) in the study were diagnosed with ASD by the psychiatrist using DSM-V (Diagnostic and Statistical Manual of Mental Disorders). Children with a history of brain trauma, seizures or psychiatric illness were excluded from the study. Furthermore, children with any systemic disorder or using any medications or enrolled in other trials were excluded from the study.

### Ethical Approval:

The ethical approval of study was obtained from Ethical Review Committee of Khyber Medical University, Peshawar (Dir/KMU-EB/SG/000760). Written informed consent was obtained. Demographic details were collected using a self-structured questionnaire. Psychometric tests, including Childhood Autism Rating Scale (CARS) and IQ scoring (Raven’s Colored Progressive Matrices) were performed. Participants received neurofeedback training thrice a week for 10 weeks (30 sessions).

### Psychometric Testing:

***Childhood Autism Rating Scale (CARS):*** It is a behavioral rating scale consisting of 15 items. Scores are determined by adding the individual ratings. Score ranges between 15 and 60. Scores between 30 and 36.5 are classified as mild to moderately autistic, while scores between 37 and 60 are classified as severely autistic.

### Neurofeedback:

Neurofeedback training was conducted utilizing neurofeedback system (BEE Medic Inc., Germany), which consisted of software Cygnet® (BEE Medic Inc., Germany) and an EEG differential amplifier EEG NeuroAmp® II (Corscience Inc., Germany). For proper conductivity, the skin at the electrode application site was prepared by cleaning with an abrasive paste and then conductive paste was used to apply Ag/AgCl electrodes. Neurofeedback training were carried out using a high-resolution gaming laptop running Windows 10 and the Cygnet System combined with Somatic Vision Video feedback. ILF-protocol and the standard method of placement of electrodes, as developed by Othmer and adopted in Othmer method was used.^3^ Infrared low frequency (ILF) neurofeedback can significantly alter functional connectivity in multiple brain areas and neural networks in ASD. Each participant received 30 separate 15-20 minutes neurofeedback sessions over 10 weeks period. Each child was evaluated using bipolar EEG montages. The electrodes were placed in accordance with the symptom profile. The electrodes were positioned at T3-T4 or T4-P4 electrode locations. The optimal reinforcement frequency (ORF) was then fine-tuned based on symptom reports from patients and parents, as well as clinical observations. The software displayed real-time EEG, and variations in amplitude were recorded. EEG amplitude denotes the power of the brain oscillations, which is measured in microvolts (μV). Mean Amplitude of δ waves in the right hemisphere is 6.1 μV, θ waves is 6.3 μV, α waves is 12.4 μV and β waves is 7.5 μV.^13^ Decrease in the exaggerated delta, theta, beta and alpha waves suggests that infra low frequency neurofeedback training can be effective in improving the brain activity.

### Statistical Analysis:

For data analysis SPSS version 25 was used. Frequency analysis was done for demographic data. Normality was checked by Shapiro-Wilk Test. Wilcoxon signed rank test was utilized for comparison of EEG before and after NFB. For gender comparison of EEG Mann Whitney U Test was utilized.

## RESULTS

The mean age of participants was 10.8±3.6 years with 68.6% males and 31.4 females. Most of the children had co-morbid illnesses where sensory sensitivities and gastrointestinal problems (14.3%) were the most reported complaints. The data reveals that 62.9% participants had mild-moderate autism and 37.1% had severe autism (Table-I). The intelligence quotient of children with ASD was assessed using Childhood Progressive Matrices (CPM). It was found that 25.7% were intellectually deficient, 22.9% were intellectually below average, 48.6% were intellectually average and 2.9% were intellectually above average. Wilcoxon signed rank test indicates that there was a significant improvement in EEG pattern after NFB therapy (Table-II). There was a significant (*p*<0.001) difference in delta waves between pre (47.31±19.22μV) and post (22.07±6.83μV) NFB intervention. Pre-intervention theta wave (24.75±16.62μV) was compared to post intervention theta wave (12.37±3.59μV) and a statistically significant (*p*<0.001) decrease in the amplitude was observed. Also, a significant (*p*<0.001) decline in the post intervention alpha waves (4.03±1.61μV) was observed when compared with the pre-intervention alpha waves (12.01±9.81μV). Beta waves also showed a significant (*p*<0.001) reduction in the post intervention amplitude (3.05±1.37μV) in comparison with the pre-intervention waves (7.61±4.26μV). Likewise, there was a significant (*p*<0.001) decrease in the post-intervention beta waves (3.41±2.46μV) in comparison with the pre- intervention (9.44±6.21μV) waves.

**Table-I:** Levels of severity of autism among children with ASD.

Levels of Severity	Frequency (f)	Percent (%)
Mild-Moderate Autistic	22	62.9
Severely Autistic	13	37.1
Total	35	100.0

**Table-II:** Comparison of EEG before and after neurofeedback training.

Brain Waves (μV)	Pre-Intervention	Post-Intervention	p value
Delta Wave	47.31 ± 19.22	22.07 ± 6.83	0.000
Theta Wave	24.75 ± 16.62	12.37 ± 3.59	0.000
Alpha Wave	12.01 ± 9.81	4.03 ± 1.61	0.000
Beta Wave	7.61 ± 4.26	3.05 ± 1.371	0.000
High Beta	9.44 ± 6.21	3.41 ± 2.46	0.000

**Fig.2:**
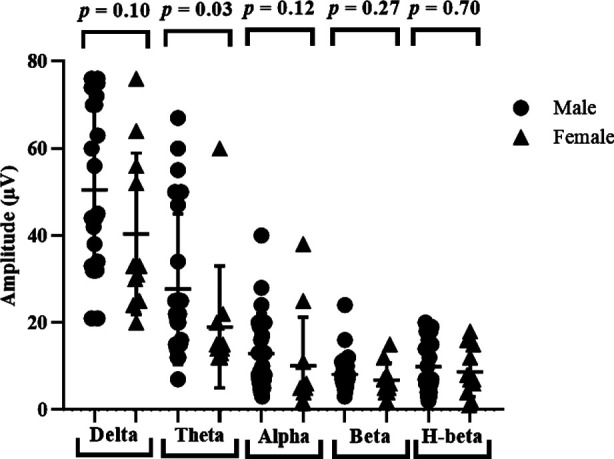
Gender comparison of brain waves (a) delta (b) theta (c) alpha (d) beta (e) high beta before neurofeedback training.

A *Mann-Whitney U test* was conducted to determine whether there is a difference in brain waves across genders or not. Fig.2 shows that male and female ASD children have no significant differences with respect to delta (U=86.50, *p*=0.105), alpha (U=89.50, *p*=0.128), beta (U=101.50, *p*=0.275) and high beta (U=121.50, *p*=0.70) waves before neurofeedback intervention. However, a significant difference between male and females have been observed in theta waves (U=73.50, *p*=0.03) before the intervention.

**Fig.3:**
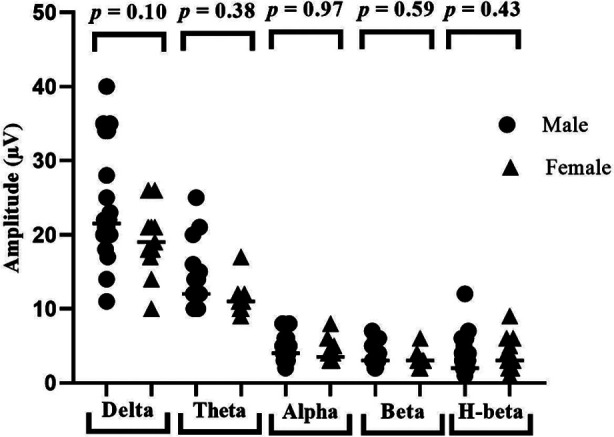
Gender comparison of brain waves (a) delta (b) theta (c) alpha (d) beta (e) high beta after neurofeedback training.

Fig.3 shows that male and female ASD children have no significant differences with respect to delta (U=86.00, *p*=0.10), theta (U=108.50, *p*=0.38), alpha (U=131.00, *p*=0.97), beta (U=117.50, *p*=0.59) and high beta (U=110, *p*=0.43) waves after neurofeedback intervention.

## DISCUSSION

Our study showed a significant decline in delta, theta, beta and alpha waves after NFB which may have a beneficial effect on the cognitive functions. No significant gender differences in EEG pattern were observed before and after NFB. However, a significant gender difference was observed in theta waves before the intervention. Relationship between resting state EEG and ASD has not been clear. Precenzano et al. found that there is an overall shift in the EEG spectrum in individuals with Autism, resulting in transformation of the power profile.^3^ Literature has not supported this pattern consistently.^3^,^9^

Our study found increased amplitude of delta waves before NFB training in ASD children, depicting decreased attention and lack of focus. NFB training aimed at reducing exaggerated delta waves. Our findings are consistent with the findings of Fauzan et al. who displayed that autistic individuals show increased delta waves.^14^ Increased delta power has been found in comparatively low-functioning children, mean IQ = 37, those with delayed mental age of > 20%, and those with 28% lower intelligence score.^9^,^15^,^16^ The increased delta power in the participants of our study may also be attributed to the intellectual capacity of the children, as 48.6% of the children were intellectually average while 22.9% had below average intellectual capacity and 25.7% were found to be intellectually defective.

In contrast to the outcomes of our study, decreased delta power has been observed in ASD children without intellectual disability. This variation may be attributed to neural underconnectivity, frontal and parietal dysfunction in autistic children.^17^ In our study, in the post intervention group delta waves were significantly decreased explaining the positive role of neurofeedback training. Studies have shown a significant decrease in delta waves after neurofeedback training.^8^,^18^An excess of delta waves during wakeful state may result in learning disabilities and may interfere with the ability to focus. Sleepwalking and talking tend to occur when delta power is increased. It disconnects the external awareness.^19^ Decrease in delta waves by NFB may play a vital role in increasing the focus of child and enhancing the mental stability.

Theta waves promote and/or mediate adaptive skills like learning and memory.^7^ Findings of our study show increased theta activity. Our findings are similar to the studies that show augmented power in theta waves.^9^,^15^ In contrast to our findings Dawson and colleagues reported a decrease in theta power in the frontal and temporal regions, but not in the parietal region.^20^ We observed frequency of theta waves decreased after neurofeedback training signifying brainwave modulations leading to improved mental attention and favorable achievement.^21^ Kouijzer et al. also found decreased theta band after NFB resulting in improved executive functions.^8^

Our study revealed that pre-intervention frequency of alpha was decreased in children with ASD which may signify impaired mirror neuron activity resulting in impaired behavior imitation and inability to mimic an instructed task.^22^ Some studies showed no variation in alpha power in children on spectrum while others exhibit increased alpha frequency bands in high-functioning ASD subjects.^17^,^23^,^24^ Findings of decreased alpha frequency band supporting the U-shaped curve model have been most consistent in low-functioning autistic children.^9^,^15^,^20^

Our findings of reduced alpha frequency band may be due to the low intellectual capacity of the children in our study. The alpha power in our study further decreased after NFB training. We measured resting state EEG while the children watched the feedback video. Our utilization of pictorial stimuli may have had a specific effect on alpha frequency band, as latest research proposes that resting states with dynamic visual images incite decreased alpha frequency relative to resting states without such stimuli.^25^ So, decreased alpha frequency may be due to the visual input presented during neurofeedback which encouraged increased interest, attention or cognitive engagement. Decreased alpha activity may also be contributed to the electrode placement as reduced alpha power has been observed across many brain regions including frontal, parietal, occipital and temporal cortex.^7^

Similar to our findings Fauzan et al. also showed an attenuation of Alpha rhythm.^15^ In contrast Kouijzer et al. found no significant change in alpha power after NFB while Oh et al. found that alpha power improved after neurofeedback training in adults.^8^,^18^ The variations among the studies may be because of the variation in neurofeedback protocols, electrode placement and sample size.

Our findings also exhibit a decrease in beta and high beta activity before neurofeedback intervention in ASD children which may account for decreased attention and difficulty in learning. Another study also showed decreased beta frequency in most of the cortical areas in ASD children which was found to be related with learning disabilities, difficulty in attention and brain injuries.^14^ Dawson and colleagues exhibited no effect in the beta band.^20^ Decreased beta frequency in the posterior region was observed during the eye open condition by Wang et al., whereas high beta failed to demonstrate any variation between eye open or closed conditions. Post intervention, in our study, the beta power did not increase rather decreased further.^7^

In contrast Kouijzer et al. found that low beta power significantly increased in ASD children after neurofeedback although a few ASD children failed to exhibit a significant surge in low beta power.^8^ Likewise, other studies also observed an increase in the frequency of beta waves after neurofeedback.^5^,^18^ In our study, neurofeedback aimed at improving the attention patterns of autistic children targeting an upsurge in beta waves but sessions could not improve the beta power. It is hypothesized that because the children got engrossed and cognitively engaged in the neurofeedback games during the intervention so the level of alertness decreased resulting in decreased beta power. Future research must be conducted to explore the effect of NFB on beta waves by changing the audiovisual stimuli, increasing the number of sessions and considering a larger sample size.

### Limitations:

As the Cygnet EEG data is not comprised of gamma band EEG, our analysis was limited to delta, theta, alpha, beta and high beta bands. Due to the patients’ narrow age range and lack of follow-up EEG, the long-term prognosis could not be provided. A substantial multicenter study with long-term follow-up is required to determine the long-term effect of ILF-NFB on EEG.

## CONCLUSION

So, Infra low frequency neurofeedback modulates EEG waves causing reintegration of the brain activity of children with ASD. EEG may serve as a biomarker for early detection in order to implement early intervention.

### Recommendations:

Since ILF-NFB targets the electrical brain activity so an early intervention may potentially contribute in improving the executive functions and quality of life of children with ASD. Additional research must be done using other study designs, a bigger representative sample, and follow-up evaluations in order to deploy NFB as a treatment modality for autistic children in clinical settings.

### Authors Contribution:

**SS:** Data Collection, Analysis & Interpretation, Drafting the manuscript and Final Approval. Responsible for Accuracy and Integrity of the study.

**SHH:** Conception & Design, Drafting the manuscript, Analysis & Interpretation and Final approval of the manuscript.
